# Neural Oscillatory and Sympathetic Arousal Dynamics During Mindfulness Meditation: Characterizing Relaxed Alertness

**DOI:** 10.1111/psyp.70425

**Published:** 2026-10-09

**Authors:** Alexander T. Duda, Adam R. Clarke, Robert J. Barry

**Affiliations:** ^1^ Brain & Behaviour Research Institute and School of Psychology University of Wollongong Wollongong New South Wales Australia

**Keywords:** EEG, mindfulness meditation, neural oscillations, relaxed alertness, skin conductance level, sympathetic arousal

## Abstract

Mindfulness meditation is often characterized as a state of relaxed alertness, yet the neural and autonomic processes that define this state remain unclear. This study examined concurrent electroencephalographic (EEG) oscillations and skin conductance level (SCL) during brief breath‐focused mindfulness meditation, with a focus on temporal dynamics. EEG and SCL were recorded during 2 min of eyes‐closed rest and a 15 min mindfulness meditation, with analyses focused on 8 min of the silent self‐guided period following instruction, divided into four consecutive 2 min segments. Planned contrasts compared eyes‐closed rest with mindfulness meditation and examined changes across the meditation period, while sex‐related variability was explored. Meditation was associated with greater theta (4–7.5 Hz) amplitude than rest. Exploratory analyses also indicated greater delta (1–3.5 Hz) amplitude during meditation and a progressive increase in delta across the meditation period. Alpha amplitude (8–13 Hz) showed no reliable state‐related or temporal effects. SCL did not differ between rest and meditation overall, although an exploratory trend analysis indicated an initial decrease followed by a later increase during meditation. Global alpha amplitude was positively associated with SCL during meditation, after accounting for participant differences and changes across the meditation period. Exploratory sex analyses suggested a larger rest‐to‐meditation theta increase in males and greater beta and gamma amplitudes in females across states, although these findings should be considered preliminary. Together, these results suggest that mindfulness meditation is not simply characterized by reduced arousal, but instead involves dynamic patterns of neural activity and sympathetic arousal that are broadly consistent with relaxed alertness.

## Impact Statement

Brief breath‐focused mindfulness meditation is characterized by increased theta activity and dynamic sympathetic arousal rather than simple physiological relaxation. By examining EEG and skin conductance concurrently across successive meditation periods, this study demonstrates that neural and autonomic responses fluctuate over time and that alpha activity relates to sympathetic arousal during meditation. These findings refine the concept of relaxed alertness and highlight the importance of temporal dynamics in meditation research.

## 1 Introduction

Over the past two decades, there has been rapid growth in research examining mindfulness, its applications, and its theoretical foundations (Baminiwatta and Solangaarachchi 2021). Mindfulness is commonly characterized as the awareness that emerges from paying attention intentionally, in the present moment, and without judgment (Kabat‐Zinn 2003). Mindfulness‐based practices have been associated with a range of health benefits, including improvements in well‐being, stress, anxiety, depression, and chronic pain (Chambers et al. 2009; Chiesa and Serretti 2010; Creswell 2017; Creswell et al. 2019; Davidson et al. 2003; Good et al. 2016; Keng et al. 2011). Despite the substantial growth of this literature, the processes through which mindfulness practice produces beneficial health outcomes remain only partially understood.

Mindfulness meditation provides a foundational means of cultivating mindfulness and includes approaches such as focused attention, open monitoring, body scanning, and mindful movement, which differ in attentional focus but share core features of mindfulness (Kabat‐Zinn 2003; Lutz et al. 2008). A central distinction in meditation research is between state and trait effects. State effects refer to immediate changes that occur during meditation, whereas trait effects describe longer‐term changes that develop through repeated meditation practice and persist beyond the meditative state (Cahn and Polich 2006; Kiken et al. 2015; Tang et al. 2015). Clarifying these acute state processes is clinically relevant because mindfulness‐based interventions are commonly used to target stress, affective symptoms, and attentional dysregulation. Neural and autonomic responses during meditation may provide mechanistic insight into the early regulatory processes through which repeated practice supports longer‐term changes in attention, arousal, and emotion regulation.

Electroencephalography (EEG), a noninvasive method for recording scalp electrical brain oscillations with high temporal resolution, has been central to investigations of the neural effects of mindfulness meditation (Barrows et al. 2024; Lieberman et al. 2025). EEG activity is commonly divided into five traditional frequency bands: delta, theta, alpha, beta, and gamma. These bands are each associated with distinct, though partially overlapping, functional processes (Knyazev 2007; Newson and Thiagarajan 2019). Although findings vary across studies, theta and alpha oscillations have received particular attention because they are among the most commonly reported state‐related EEG changes during meditation and are closely associated with attentional regulation, inhibitory control, awareness, and arousal modulation (Aftanas and Golocheikine 2001; Baijal and Srinivasan 2010; Cahn et al. 2010, 2013; Cooper et al. 2003; Jensen and Mazaheri 2010; Klimesch 2007, 2012; Lagopoulos et al. 2009). By contrast, delta, beta, and gamma oscillations have been examined less consistently in mindfulness meditation research, although some evidence suggests that they may also contribute to meditation‐related neural changes (Deolindo et al. 2020; Lee et al. 2018). Overall, variability in meditation style, participant experience, study design, EEG methodology, and outcome measures continues to limit clear conclusions about the neural state effects of mindfulness meditation (Barrows et al. 2024; Davidson and Kaszniak 2015; Lomas et al. 2015; Van Dam et al. 2018).

Arousal has been proposed as an important component of the meditative state, with mindfulness meditation often characterized as a state of “relaxed alertness” involving sustained attentional engagement within a calm but wakeful physiological state (Britton et al. 2014; Lomas et al. 2015). This view suggests that mindfulness meditation is not simply a passive relaxation state, but an active state in which attention and arousal may interact in complex ways. Skin conductance level (SCL), an index of sympathetic nervous system activity, is widely used to assess physiological arousal (Barry and Sokolov 1993; Barry et al. 2005, 2020; Figner and Murphy 2011), but remains underutilized relative to EEG in mindfulness research. Examining SCL alongside neural oscillatory activity may therefore provide greater insight into how sympathetic arousal is regulated during meditation and how this relates to the neural processes supporting the meditative state.

This is particularly relevant for alpha and theta oscillations, which have been among the most consistently reported EEG changes during mindfulness meditation. Relative to eyes‐closed rest, increases in alpha and theta have been reported most frequently, with their co‐occurrence proposed to reflect a state of relaxed alertness, although these effects are not uniformly observed across studies (Lomas et al. 2015). However, neural oscillatory changes alone provide limited information about the accompanying physiological arousal state, highlighting the value of adding a direct index of sympathetic activity such as SCL. Within this context, the relationship between alpha activity and sympathetic arousal is particularly relevant. During rest, global alpha amplitude has been inversely associated with SCL, such that greater alpha activity accompanies lower sympathetic arousal (Barry et al. 2005, 2020). In contrast, studies examining concurrent EEG and SCL during mindfulness meditation suggest that meditation‐related alpha changes are not necessarily accompanied by reductions in SCL and may, in some contexts, be positively associated with SCL (Duda et al. 2024, 2026; Duda, Clarke, and Barry 2025; Duda, Clarke, Barry, and De Blasio 2025). Together, these findings suggest that the relationship between alpha activity and sympathetic arousal during mindfulness meditation may differ from that typically observed during resting states.

Although studies often treat meditation as a single, uniform state, recent evidence suggests that neural oscillations can change dynamically across the course of a meditation session, with EEG changes emerging within minutes and varying over time during meditation (Saketh et al. 2026). This aligns with the view that meditation is an active and fluctuating state, requiring ongoing regulation of attention, awareness of distraction, and reorientation to the object of meditation. Consistent with this view, breath‐focused meditation studies have shown within‐session differences in theta and alpha oscillations during episodes of mind‐wandering relative to breath focus (Rodriguez‐Larios et al. 2021), while breath‐counting research has shown that mind‐wandering is associated with both reduced alpha oscillations and increased SCL relative to breath focus (Rout et al. 2024). Such temporal variability may help explain inconsistencies in the literature, as averaging across an entire meditation period may obscure meaningful changes in neural and physiological activity. Together, these findings suggest that meditation is not static, but involves dynamic changes that unfold over time.

Individual differences may further contribute to variability in neural and autonomic responses during mindfulness meditation. Sex may be one source of psychophysiological variability, with differences in resting EEG profiles well documented (Cave and Barry 2021), while some evidence suggests that meditation‐related outcomes may also differ between males and females (Rojiani et al. 2017). Hormonal factors may further contribute to this variability, with menstrual‐cycle phase associated with differences in resting EEG activity, particularly within the alpha range (Bazanova et al. 2014, 2017; Brötzner et al. 2014). Given the prominent role of alpha and theta oscillations in mindfulness meditation, together with their proposed relationships with arousal and attentional processes, sex‐related variability may therefore be relevant to understanding individual differences in the meditative state. Accordingly, sex‐related differences in EEG and SCL responses were explored here.

The present study primarily sought to characterize the neural and sympathetic features of relaxed alertness during brief breath‐focused mindfulness meditation. Relaxed alertness was operationalized as increased theta and/or alpha activity relative to eyes‐closed rest, consistent with continued attentional engagement, in the absence of a marked sustained shift in SCL. We first compared rest and meditation to assess this pattern, and then explored changes across the four consecutive meditation segments to determine whether these physiological responses remained stable or changed over time. We also examined whether global alpha amplitude was associated with SCL during meditation. Additional exploratory analyses examined delta, beta, and gamma activity and sex‐related variability.

## 2 Method

### 2.1 Participants

A sex‐balanced sample of 42 participants aged 18–35 years was included in this study: 21 females (M_age_ = 20.7, SD = 3.3 years) and 21 males (M_age_ = 21.7, SD = 4.5 years). Sex was recorded as self‐reported female or male. The term “sex” is used throughout to refer to this self‐reported classification. Written informed consent was obtained from all participants prior to commencement of the study. This study received approval from the joint University of Wollongong/Illawarra Shoalhaven Local Health District Human Research Ethics Committee (2020/363).

Participants reported no history of neurological or psychiatric conditions, no history of serious head injury, and normal hearing, and were right‐handed as confirmed by the Edinburgh Handedness Inventory Short Form (Veale 2014). They were fluent in English. Prior to the experimental session, participants were instructed to abstain from alcohol, caffeine, tobacco, and psychoactive medications or substances for at least 4 h, which was confirmed at the commencement of the experimental session. All participants had limited lifetime meditation experience, ranging from 0 to 50 h (M_exp_ = 11.1, SD = 14.8 h), and were classified within the beginner meditation‐experience category (0–100 h) described by Van Dam et al. (2024).

### 2.2 Power Analysis

Sensitivity analyses were conducted in G*Power 3.1 (Faul et al. 2009) using a repeated‐measures multivariate analysis of variance (MANOVA) with a within–between interaction to determine the minimum detectable effect size for the final sex‐balanced sample. The analysis was specified for the interaction between sex and the rest‐to‐meditation contrast, with α = 0.05 and power = 0.80. The final sample of *N* = 42 was sensitive to large interaction effects, with a minimum detectable effect size of *f*(V) = 0.44.

### 2.3 Procedure

Upon arrival at the experimental session, participants received an information sheet, provided written informed consent, and completed questionnaires not analyzed in the present report. After eligibility was confirmed, participants were fitted with the physiological recording equipment. Participants were then moved to a dimly lit, air‐conditioned room, where they sat comfortably upright in a chair positioned 80 cm from a 17‐in. monitor for the remainder of the session. The first task involved a 3 min electrooculogram (EOG) calibration to measure vertical and horizontal eye movements and blinks (Croft and Barry 2000), followed by 2 min of eyes‐closed rest, 2 min of eyes‐open rest, a second 2 min of eyes‐closed rest, and a 15 min mindfulness meditation.

Only the second eyes‐closed rest period was used as the resting baseline because it immediately preceded meditation, providing the temporally closest estimate of each participant's premeditation physiological state after acclimatization to the recording environment.

### 2.4 Physiological Recording

EEG data were recorded using a 19‐channel tin electrode cap (Fp1, Fp2, F7, F3, Fz, F4, F8, T7, C3, Cz, C4, T8, P7, P3, Pz, P4, P8, O1, O2) arranged according to the International 10–20 System, with AFz as ground. EEG was referenced online to the left earlobe (A1), and the right earlobe (A2) was recorded as an active electrode to allow offline re‐referencing. EOG activity was recorded from electrodes placed above (E2) and below (E4) the right eye and at the outer canthi of the left (E5) and right (E6) eyes. Electrode impedances were maintained below 5 kΩ and monitored throughout recording.

SCL data were recorded using two silver/silver chloride (Ag/AgCl) electrodes attached to the distal phalanges of the index and middle fingers of the nondominant (left) hand. The electrodes were filled with an electrolyte consisting of 0.05 M NaCl in an inert ointment base and connected to a constant‐voltage device (UFI BioDerm 2701) set at 0.5 V.

EEG and SCL data were acquired synchronously using a Neuroscan SynAmps2 amplifier and Acquire software (Compumedics, version 4.5.1), with a sampling rate of 500 Hz, a bandwidth of DC–70 Hz, and a 50 Hz notch filter.

### 2.5 Mindfulness Meditation

The mindfulness meditation was adapted from *Mindfulness for Beginners: Mindfulness of Breathing* (Kabat‐Zinn 2006). Participants received a brief overview of the meditation and were given the opportunity to ask questions before beginning. They were then instructed to sit comfortably, close their eyes, and focus on their breath. The meditation lasted 15 min and was delivered through Sony MDR‐V700 headphones, comprising 6 min of audio guidance followed by 9 min of silent, self‐directed meditation. The audio guidance emphasized maintaining awareness of the breath as an anchor of attention and adopting a nonjudgmental attitude toward ongoing experience. When attention wandered, participants were encouraged to notice this and gently return their attention to the breath. Only the 8 min of the silent period immediately following the audio guidance were included in the present analyses. The final minute was excluded to retain four equal‐duration segments.

### 2.6 Data Quantification

All data processing was conducted in MATLAB R2024b using custom scripts and EEGLAB (v2025.0) (Delorme and Makeig 2004). EEG data were first corrected for ocular artifacts using the Revised Aligned‐Artifact Average procedure (Croft and Barry 2000). The EOG‐corrected data were then digitally re‐referenced to the average earlobes. Data were visually inspected for bad channels, which were interpolated in EEGLAB using spherical spline interpolation (M = 1.3, SD = 1.2 channels). EEG data were extracted from the second 2 min eyes‐closed rest (EC) and from four 2 min segments of mindfulness meditation (MM): 6–8 min (MM1), 8–10 min (MM2), 10–12 min (MM3), and 12–14 min (MM4). Each 2 min segment was divided into 60 sequential, nonoverlapping 2 s epochs, with each epoch baseline‐corrected using its mean voltage. Epochs were automatically rejected if any channel contained extreme amplitudes (±300 μV), abrupt voltage changes (> 50 μV between consecutive data points), or flatlined activity (range < 0.5 μV over 100 ms). A 10% Hanning window was then applied to the remaining epochs, followed by a discrete Fourier transform to derive single‐sided amplitude spectra with a frequency resolution of 0.5 Hz. To account for windowing effects, amplitudes were multiplied by 1.0528 and then averaged across epochs to obtain the EEG spectra. Absolute spectral amplitudes were calculated for the delta (1.0–3.5 Hz), theta (4.0–7.5 Hz), alpha (8.0–13.0 Hz), beta (13.5–30.0 Hz), and gamma (30.5–45.0 Hz) bands by summing the corresponding 0.5 Hz frequency bins for each electrode. These values were then averaged across the 19 scalp electrodes to obtain a global amplitude (μV) for each participant in each state (EC, MM1, MM2, MM3, and MM4) for subsequent analyses.

SCL data were extracted from the second 2 min eyes‐closed rest period (EC) and from four consecutive 2 min mindfulness meditation segments: 6–8 min (MM1), 8–10 min (MM2), 10–12 min (MM3), and 12–14 min (MM4). Each 2 min segment was divided into 60 sequential, nonoverlapping 2 s epochs. The SCL data were visually inspected for artifacts, and mean SCL (μS) was calculated for each participant in each state (EC, MM1, MM2, MM3, and MM4) for subsequent analyses.

### 2.7 Statistical Analyses

#### 2.7.1 EEG and SCL

Analyses were performed using IBM SPSS Statistics (v26). Prior to analysis, data were screened for outliers and assessed for normality. Univariate outliers were defined as absolute *z* scores exceeding 2.576 (two‐tailed *p* = 0.01) within each combination of state and sex. Outliers were identified in 1.0% of the EEG data and were winsorized by replacing each value with the next less extreme value and adjusting it by 0.01 in the direction of the original outlier, thereby preserving its status as the most extreme value (Tabachnick and Fidell 2013). No outliers were identified in the SCL data.

For each EEG band and SCL separately, a mixed repeated‐measures analysis was conducted using the MANOVA procedure with multivariate tests for the repeated factor, with sex (Female, Male) as the between‐subjects factor and state (EC, MM1, MM2, MM3, MM4) as the within‐subjects factor. Special orthogonal contrasts were specified for state to compare EC with the mean of MM1–MM4 and to examine linear, quadratic, and cubic trends across MM1–MM4. The rest‐to‐meditation contrasts for theta, alpha, and SCL, together with their linear changes across meditation, were treated as focal analyses within the relaxed‐alertness framework. Quadratic and cubic temporal contrasts, delta, beta, and gamma analyses, and all sex‐related effects were interpreted as exploratory.

#### 2.7.2 EEG–SCL Association

To examine within‐participant associations between global alpha amplitude and SCL during mindfulness meditation, alpha amplitude was decomposed into within‐ and between‐participant components across MM1–MM4. For each participant, Alpha_mean_ represented mean alpha amplitude across the four meditation segments, while Alpha_within_ represented each segment's deviation from that participant's mean. Alpha_between_ represented the participant's mean alpha amplitude minus the sample grand mean of participants' mean alpha amplitudes. Meditation segment was included as a categorical fixed effect, and participant was included as a random intercept.

The primary model was specified as:

$$\mathrm{SCL}\sim {\text{Alpha}}_{\text{within}}+{\text{Alpha}}_{\text{between}}+\text{Segment}+\left(1\;|\;\text{Participant}\right)$$

The exploratory sex‐moderation model was specified as:

$$\mathrm{SCL}\sim {{\text{Alpha}}_{\text{within}}}^{*}\;\mathrm{Sex}+{\text{Alpha}}_{\text{between}}+\text{Segment}+\left(1\;|\;\text{Participant}\right)$$

SCL was specified as the outcome to provide a common autonomic measure across EEG‐band models. The exploratory sex‐moderation model tested the Alpha_within_ by Sex interaction, with females as the reference group. Exploratory analyses examined whether corresponding associations were also present for delta, theta, beta, and gamma by repeating the primary model with the corresponding within‐ and between‐participant terms substituted for alpha.

Models were estimated in R (Version 4.6.1; R Core Team 2026) using the lme4 and lmerTest packages (Bates et al. 2015; Kuznetsova et al. 2017). Restricted maximum likelihood estimation was used, with Satterthwaite degrees of freedom for fixed‐effect tests, and 95% Wald confidence intervals were calculated for fixed‐effect estimates. Model assumptions were assessed by visual inspection of residual‐versus‐fitted plots for linearity and homoscedasticity, Q‐Q plots of conditional residuals, and Q‐Q plots of participant random intercepts.

## 3 Results

### 3.1 EEG

The number of accepted EEG epochs was comparable across states and sexes, ranging from 52 to 60 epochs (M = 59.3, SD = 1.5 epochs). Figure 1 displays the spectral distributions of the global grand mean amplitudes for eyes‐closed rest and mindfulness meditation, separately for each sex, with EEG frequency bands indicated. Across all plots, the spectral profiles followed the expected pattern: a delta peak, a gradual decline through theta, an alpha peak, and a subsequent decline across the beta and gamma ranges.

**FIGURE 1:**
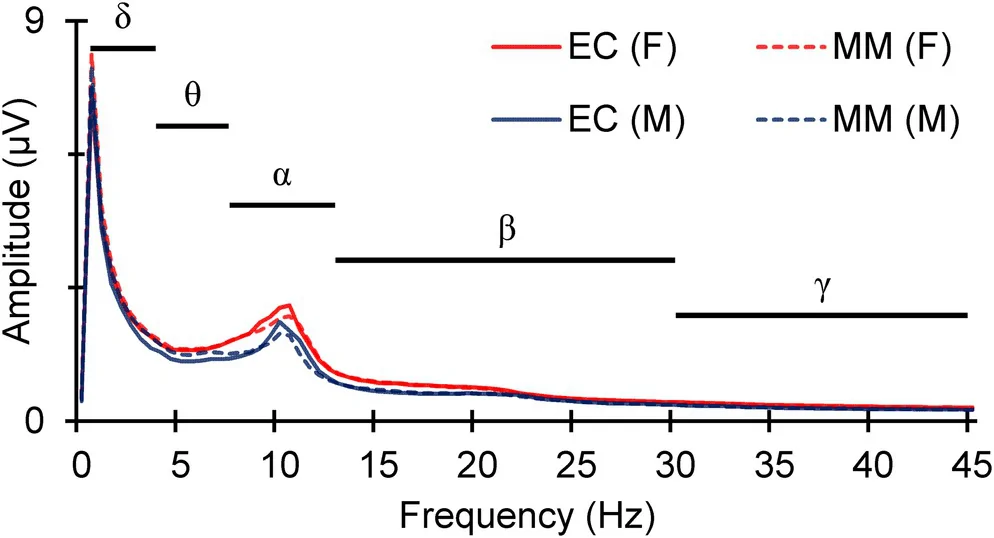
Global grand mean spectral amplitudes for eyes‐closed rest (EC; solid lines) and mindfulness meditation (MM; dashed lines), shown separately for females (F; red) and males (M; blue). EEG frequency bands are indicated on the figure: δ = delta (1.0–3.5 Hz), θ = theta (4.0–7.5 Hz), α = alpha (8.0–13.0 Hz), β = beta (13.5–30.0 Hz), and γ = gamma (30.5–45.0 Hz).

#### 3.1.1 Delta

Delta amplitude appeared maximal along the midline across states and was slightly more anterior in males (Figure 2A). In exploratory analyses, global delta amplitude was significantly greater during mindfulness meditation than eyes‐closed rest, *F*(1, 40) = 16.46, *p* < 0.001, *η*_p_^2^ = 0.29, 95% CI [0.08, 0.49] (Figure 2B). Within mindfulness meditation, a significant linear trend indicated a progressive increase in global delta amplitude from MM1 to MM4, *F*(1, 40) = 4.52, *p* = 0.040, *η*_p_^2^ = 0.10, 95% CI [0.00, 0.30] (Figure 2C). No other significant delta effects were observed (all *p* ≥ 0.051).

**FIGURE 2:**
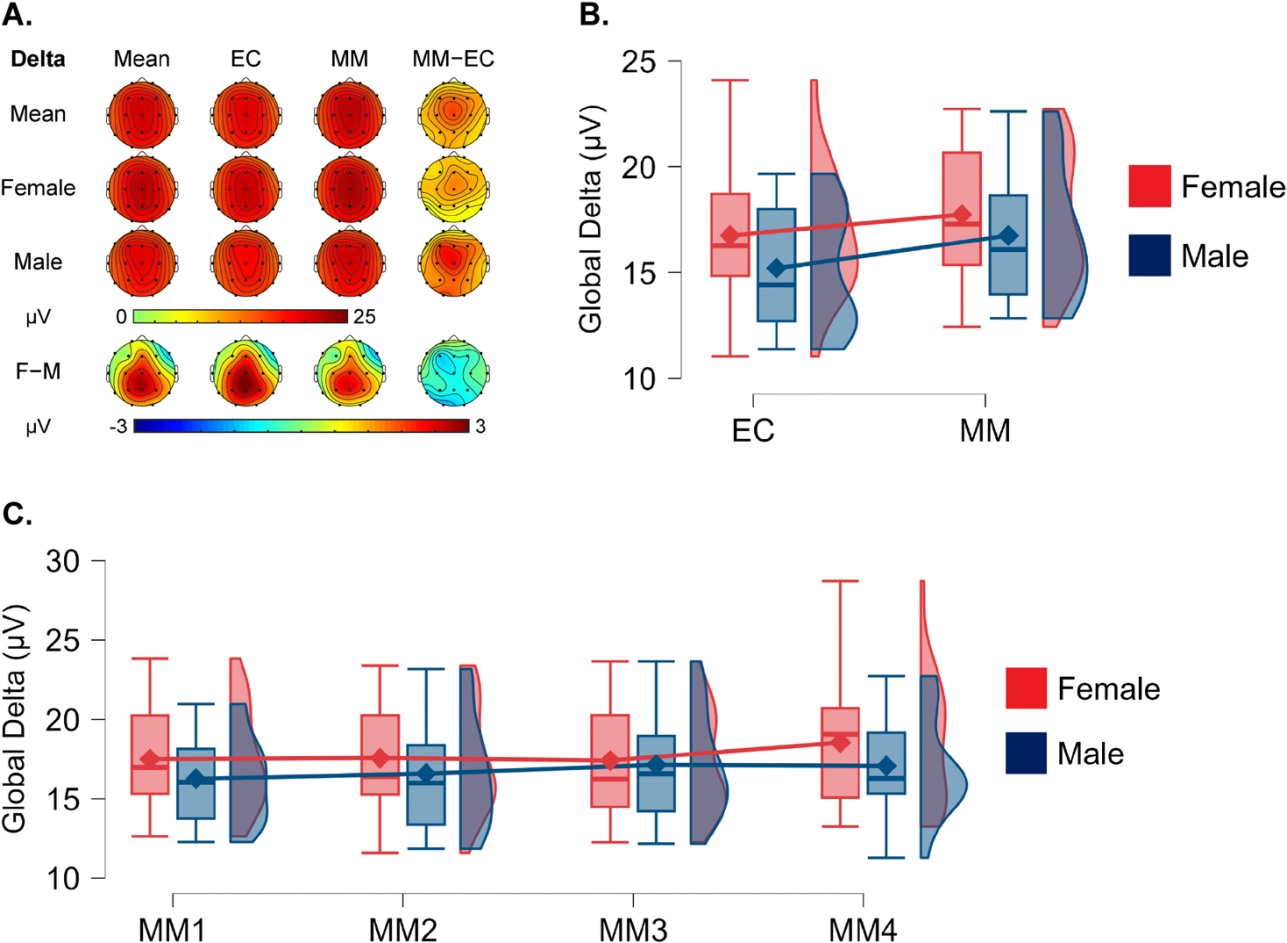
Delta amplitude (1.0–3.5 Hz) during eyes‐closed rest and mindfulness meditation. (A) Scalp topographies showing mean delta amplitude during eyes‐closed rest (EC), mindfulness meditation (MM), and the MM–EC difference, displayed for the overall mean, females (F), males (M), and the female–male difference (F–M). (B) Boxplots of global delta amplitude during EC and MM, shown separately for females and males. (C) Boxplots of global delta amplitude across the four mindfulness meditation segments (MM1, MM2, MM3, and MM4). In (B) and (C), diamonds indicate means and lines connect sex‐specific means. Red = female; blue = male.

#### 3.1.2 Theta

Theta amplitude appeared maximal along the midline across states, with a modest anterior shift in males (Figure 3A). Global theta amplitude was significantly greater during mindfulness meditation than eyes‐closed rest, *F*(1, 40) = 14.05, *p* = 0.001, *η*_p_^2^ = 0.26, 95% CI [0.06, 0.46] (Figure 3B). An exploratory state by sex interaction was also observed, *F*(1, 40) = 4.09, *p* = 0.050, *η*_p_^2^ = 0.09, 95% CI [0.00, 0.29], with males showing a larger increase from eyes‐closed rest to mindfulness meditation than females. No other significant theta effects were observed (all *p* ≥ 0.062).

**FIGURE 3:**
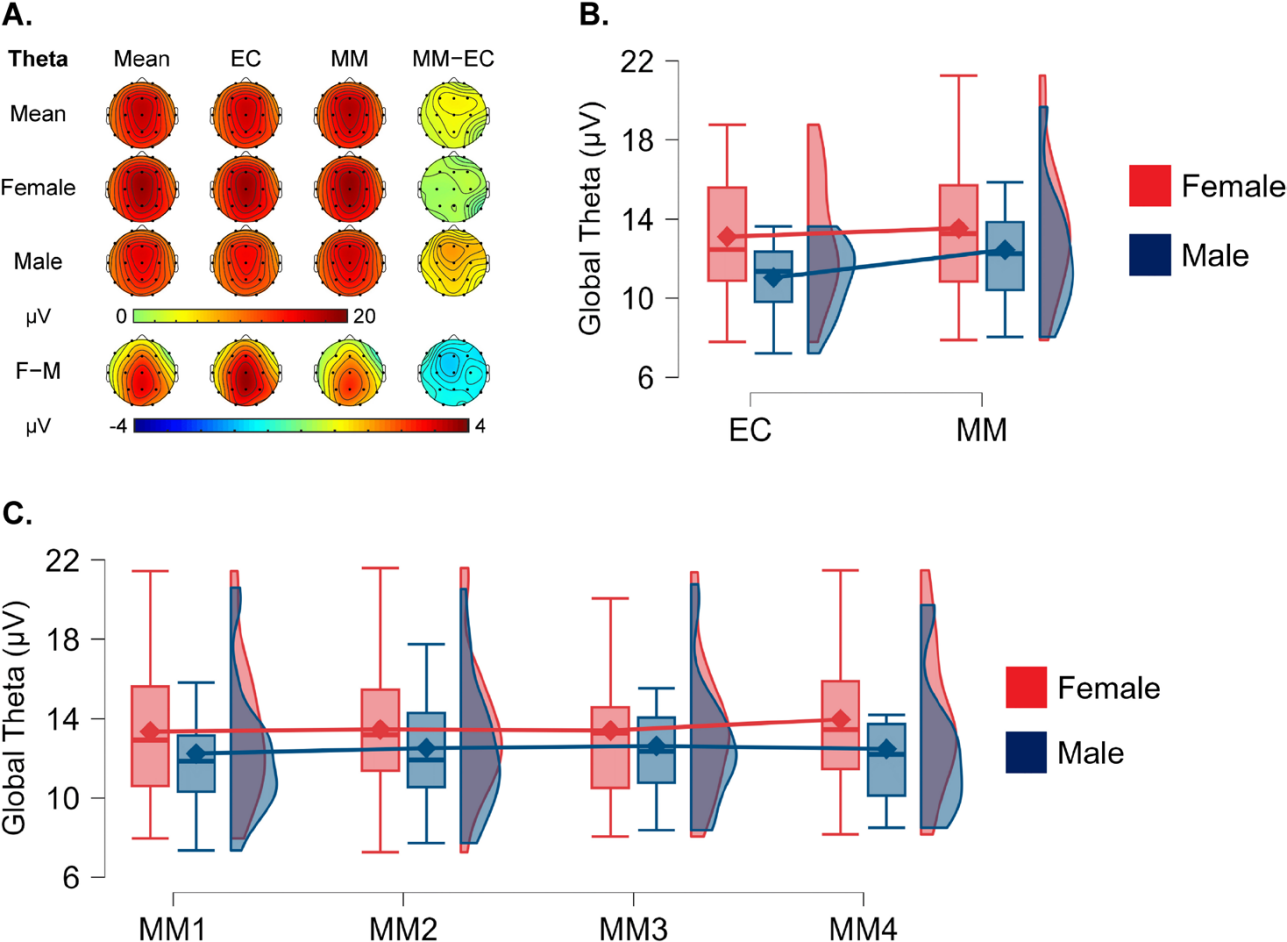
Theta amplitude (4.0–7.5 Hz) during eyes‐closed rest and mindfulness meditation. (A) Scalp topographies showing mean theta amplitude during eyes‐closed rest (EC), mindfulness meditation (MM), and the MM–EC difference, displayed for the overall mean, females (F), males (M), and the female–male difference (F–M). (B) Boxplots of global theta amplitude during EC and MM, shown separately for females and males. (C) Boxplots of global theta amplitude across the four mindfulness meditation segments (MM1, MM2, MM3, and MM4). In (B) and (C), diamonds indicate means and lines connect sex‐specific means. Red = female; blue = male.

#### 3.1.3 Alpha

Alpha amplitude appeared maximal over posterior regions across states in both sexes (Figure 4A). Global alpha amplitude did not differ significantly between eyes‐closed rest and mindfulness meditation, *F*(1, 40) = 2.58, *p* = 0.116, *η*_p_^2^ = 0.06, 95% CI [0.00, 0.24] (Figure 4B). No other significant alpha effects were observed (all *p* ≥ 0.068).

**FIGURE 4:**
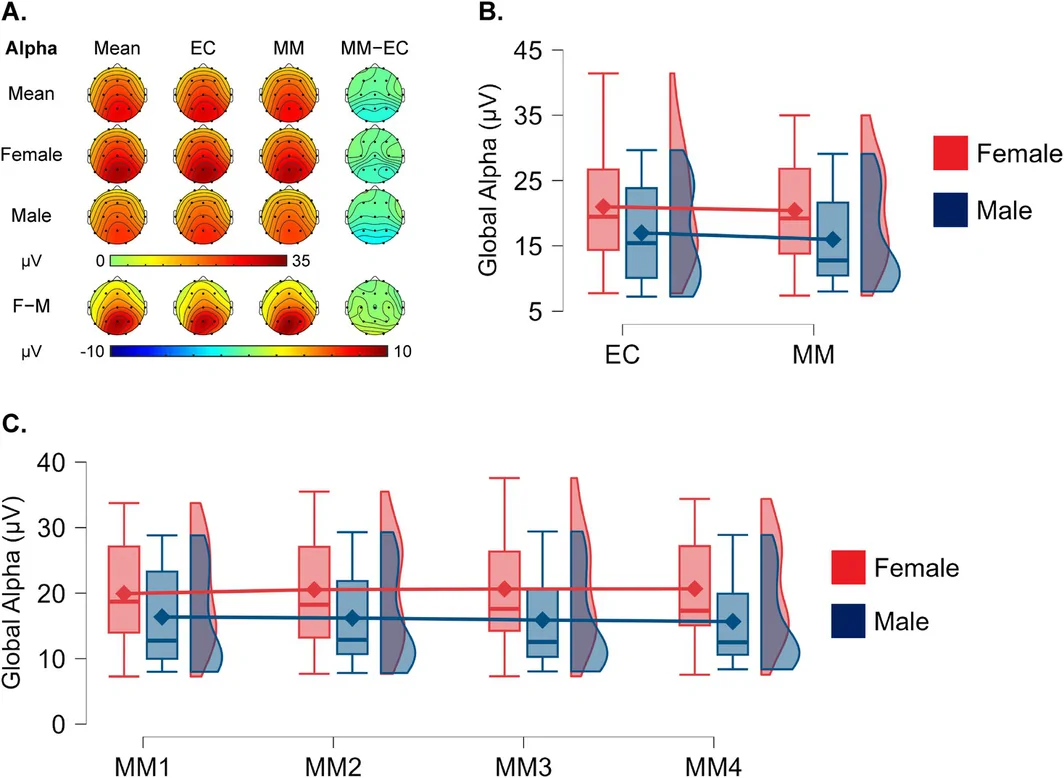
Alpha amplitude (8.0–13.0 Hz) during eyes‐closed rest and mindfulness meditation. (A) Scalp topographies showing mean alpha amplitude during eyes‐closed rest (EC), mindfulness meditation (MM), and the MM–EC difference, displayed for the overall mean, females (F), males (M), and the female–male difference (F–M). (B) Boxplots of global alpha amplitude during EC and MM, shown separately for females and males. (C) Boxplots of global alpha amplitude across the four mindfulness meditation segments (MM1, MM2, MM3, and MM4). In (B) and (C), diamonds indicate means and lines connect sex‐specific means. Red = female; blue = male.

#### 3.1.4 Beta

Beta amplitude appeared maximal at the posterior midline in females across states and along the midline in males (Figure 5A). In exploratory analyses, global beta amplitude was greater in females than in males across states, *F*(1, 40) = 8.67, *p* = 0.005, *η*_p_^2^ = 0.18, 95% CI [0.02, 0.38] (Figure 5B). Within mindfulness meditation, an exploratory sex by cubic trend interaction was observed, *F*(1, 40) = 4.30, *p* = 0.045, *η*_p_^2^ = 0.10, 95% CI [0.00, 0.29], with females showing an initial increase at MM2, followed by a decrease at MM3 and a later increase at MM4, whereas the male mean increased to MM3 and decreased slightly at MM4 (Figure 5C). No other significant beta effects were observed (all *p* ≥ 0.173).

**FIGURE 5:**
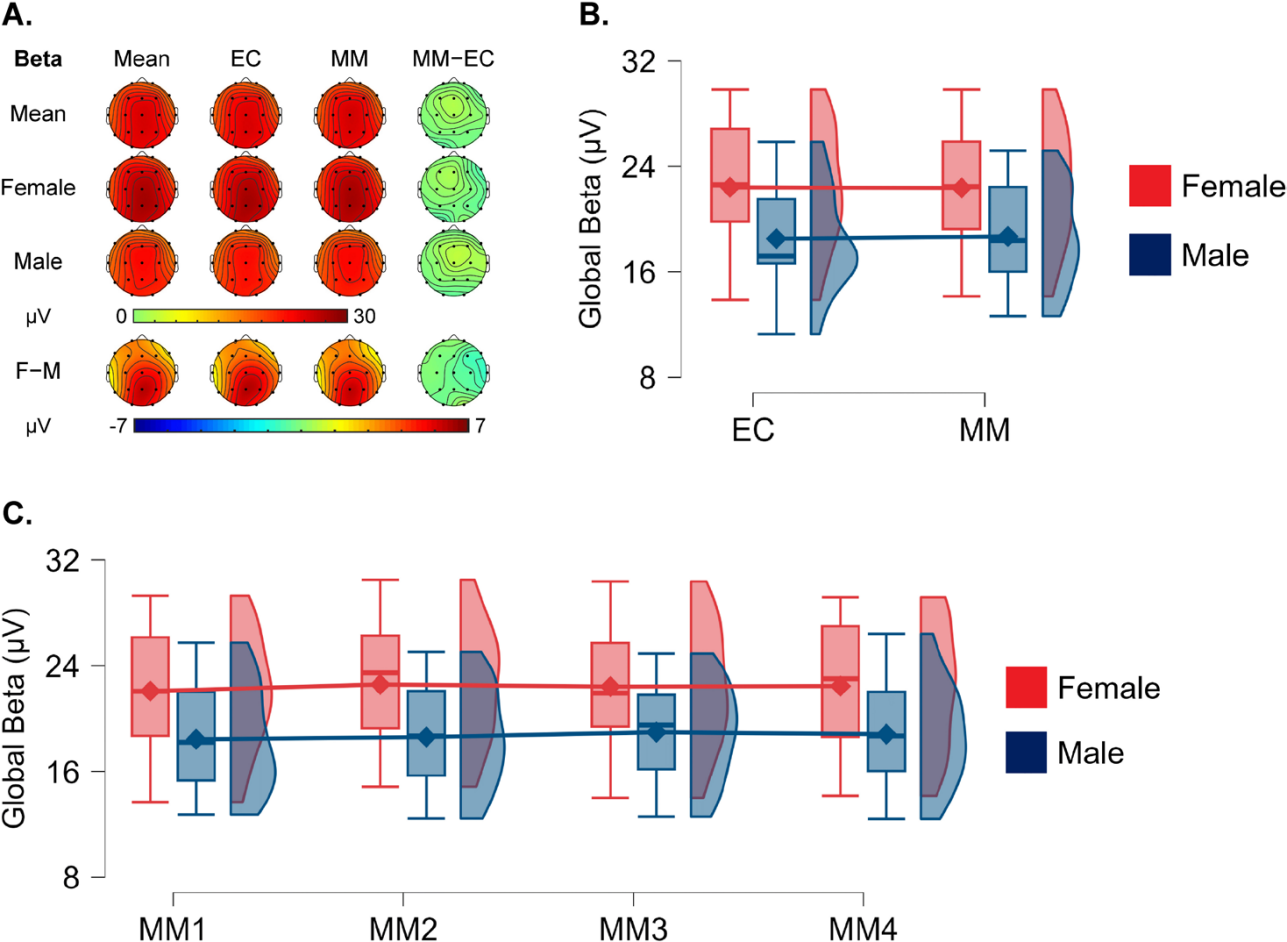
Beta amplitude (13.5–30.0 Hz) during eyes‐closed rest and mindfulness meditation. (A) Scalp topographies showing mean beta amplitude during eyes‐closed rest (EC), mindfulness meditation (MM), and the MM–EC difference, displayed for the overall mean, females (F), males (M), and the female–male difference (F–M). (B) Boxplots of global beta amplitude during EC and MM, shown separately for females and males. (C) Boxplots of global beta amplitude across the four mindfulness meditation segments (MM1, MM2, MM3, and MM4). In (B) and (C), diamonds indicate means and lines connect sex‐specific means. Red = female; blue = male.

#### 3.1.5 Gamma

Gamma amplitude appeared diffuse across the scalp across states (Figure 6A). In exploratory analyses, global gamma amplitude was greater in females than males across states, *F*(1, 40) = 6.41, *p* = 0.015, *η*_p_^2^ = 0.14, 95% CI [0.00, 0.34] (Figure 6B). No other significant gamma effects were observed (all *p* ≥ 0.067).

**FIGURE 6:**
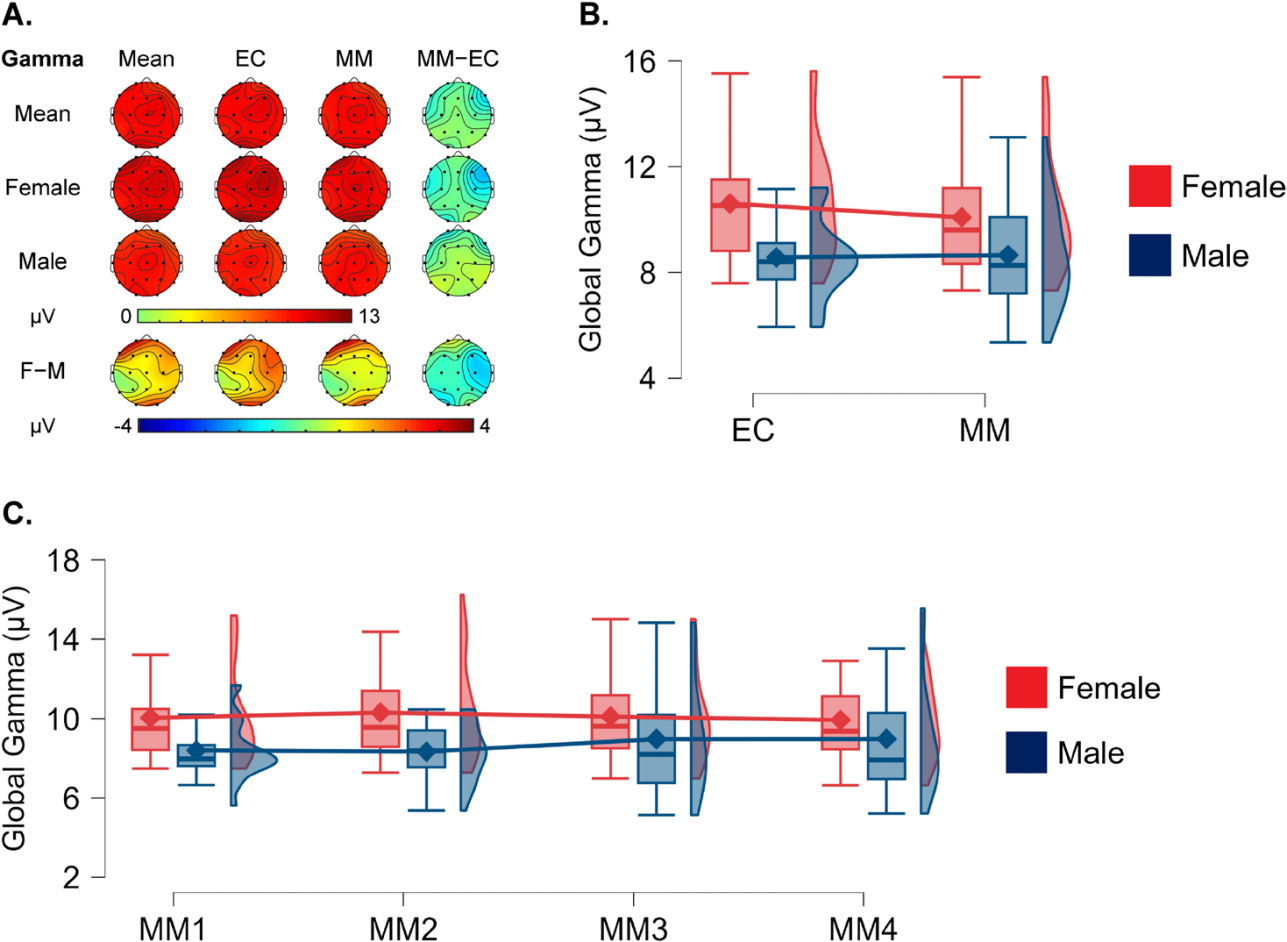
Gamma amplitude (30.5–45.0 Hz) during eyes‐closed rest and mindfulness meditation. (A) Scalp topographies showing mean gamma amplitude during eyes‐closed rest (EC), mindfulness meditation (MM), and the MM–EC difference, displayed for the overall mean, females (F), males (M), and the female–male difference (F–M). (B) Boxplots of global gamma amplitude during EC and MM, shown separately for females and males. (C) Boxplots of global gamma amplitude across the four mindfulness meditation segments (MM1, MM2, MM3, and MM4). In (B) and (C), diamonds indicate means and lines connect sex‐specific means. Red = female; blue = male.

### 3.2 SCL

SCL did not differ significantly between eyes‐closed rest and mindfulness meditation, *F*(1, 40) = 1.22, *p* = 0.276, *η*_p_^2^ = 0.03, 95% CI [0.00, 0.19] (Figure 7). Within mindfulness meditation, an exploratory quadratic contrast indicated an initial decrease in SCL followed by a later increase, *F*(1, 40) = 5.69, *p* = 0.022, *η*_p_^2^ = 0.12, 95% CI [0.00, 0.33]. No other significant SCL effects were observed (all *p* ≥ 0.160).

**FIGURE 7:**
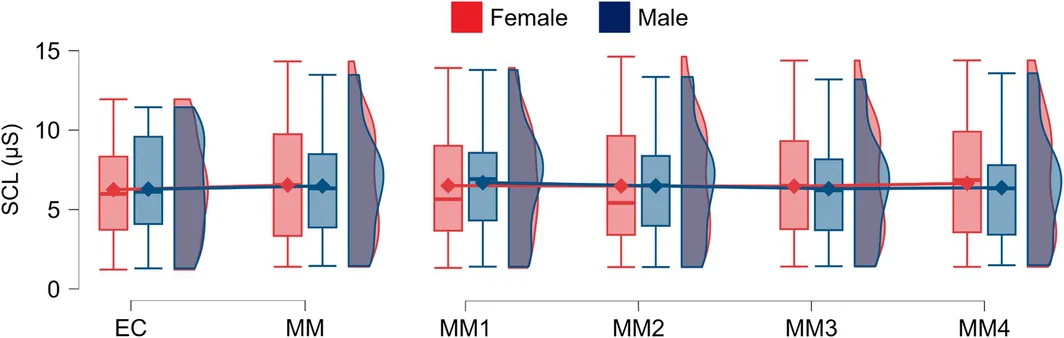
Skin conductance level (SCL) during eyes‐closed rest (EC) and mindfulness meditation (MM). Boxplots show mean SCL for EC, MM, and the four mindfulness meditation segments (MM1, MM2, MM3, and MM4), shown separately for females and males. Diamonds indicate means, and lines connect sex‐specific means. Red = female; blue = male.

### 3.3 EEG–SCL Association

Within participants, greater global alpha amplitude relative to a participant's own mean was associated with higher concurrent SCL after accounting for meditation segment and between‐participant alpha differences, *b* = 0.080, SE = 0.025, 95% CI [0.032, 0.129], *p* = 0.001. No corresponding within‐participant association was observed for delta, *b* = 0.038, SE = 0.027, 95% CI [−0.014, 0.090], *p* = 0.155; theta, *b* = −0.005, SE = 0.044, 95% CI [−0.091, 0.082], *p* = 0.918; beta, *b* = 0.014, SE = 0.043, 95% CI [−0.071, 0.099], *p* = 0.751; or gamma amplitude, *b* = 0.013, SE = 0.037, 95% CI [−0.059, 0.085], *p* = 0.727.

In an exploratory sex‐moderation model, the estimated within‐participant alpha–SCL association was positive in females (*b* = 0.113, SE = 0.029, 95% CI [0.055, 0.170], *p* < 0.001) and near zero in males (*b* = 0.008, SE = 0.044, 95% CI [−0.078, 0.095], *p* = 0.852). However, the Alpha_within_ by Sex interaction did not reach significance (*b* = −0.104, SE = 0.054, 95% CI [−0.209, 0.001], *p* = 0.053).

## 4 Discussion

The present findings characterize brief breath‐focused mindfulness meditation as a dynamic neurophysiological state involving changes in both neural oscillatory activity and sympathetic arousal. Relative to eyes‐closed rest, meditation was associated with greater theta amplitude, while SCL showed no marked overall shift between states. Alpha amplitude did not differ reliably between rest and meditation, but was positively associated with SCL during meditation. Exploratory analyses also indicated a progressive increase in delta amplitude and an initial decrease followed by a later increase in SCL during meditation. Together, these findings are broadly consistent with relaxed alertness, in which attentional engagement is maintained without a corresponding sustained increase or decrease in sympathetic arousal.

The increase in theta provided the clearest neural distinction between meditation and eyes‐closed rest in the present data. Theta enhancement is among the more consistently reported EEG findings in mindfulness research and has been linked with internally directed attention, monitoring, cognitive control, and awareness of ongoing experience (Aftanas and Golocheikine 2001; Baijal and Srinivasan 2010; Karakaş 2020). These processes are particularly relevant to focused‐attention meditation, where attention is maintained on an object such as the breath, distractions are monitored, and attention is repeatedly redirected when required (Lutz et al. 2008; Tang et al. 2015). The greater theta amplitude observed here is therefore consistent with the attentional demands of breath‐focused mindfulness rather than a passive reduction in neural activity. Importantly, this change occurred without a corresponding overall shift in SCL, suggesting that increased attentional engagement was not accompanied by a marked change in sympathetic arousal.

The SCL findings further indicate that sympathetic arousal was not simply reduced during meditation. Although average SCL did not differ between eyes‐closed rest and meditation, SCL decreased initially before increasing later in the meditation period. Rather than reflecting a uniform movement toward lower arousal, this pattern suggests that sympathetic activity fluctuated across practice. Such variability is compatible with accounts of meditation in which arousal is regulated to support continued wakefulness and attentional engagement rather than progressively suppressed (Britton et al. 2014; Takahashi et al. 2005). Previous studies combining EEG and autonomic measures similarly indicate that meditation‐related changes in neural activity need not be accompanied by parallel reductions in sympathetic arousal (Duda et al. 2024, 2026; Duda, Clarke, and Barry 2025; Duda, Clarke, Barry, and De Blasio 2025; Takahashi et al. 2005). As SCL reflects sympathetic activity rather than autonomic activity more broadly (Figner and Murphy 2011), these findings do not establish a particular autonomic state. They do, however, suggest that mindfulness meditation can involve a regulated, engaged state without a sustained reduction in sympathetic arousal.

Alpha amplitude did not reliably distinguish meditation from the eyes‐closed resting state. Although increases in alpha activity are commonly reported during meditation, findings vary according to meditation style, practitioner experience, comparison condition, and analytic approach (Deolindo et al. 2020; Lee et al. 2018; Lomas et al. 2015). The comparison condition is particularly relevant here, as eye closure itself produces prominent changes in alpha activity (Barry et al. 2007). Detecting an additional meditation‐related increase may therefore be more difficult against an eyes‐closed baseline than against an eyes‐open or otherwise less closely matched state. The absence of a reliable alpha increase may also partly reflect the relative inexperience of the present sample, as meditation‐related alpha activity has been shown to differ according to practitioner experience and proficiency (Kakumanu et al. 2018). Accordingly, the present findings should be interpreted as a lack of reliable change in global alpha activity, rather than evidence that alpha was uninvolved in meditation. Global measures may also obscure spatial variation, individual differences in alpha frequency, or functionally distinct alpha components (Barry et al. 2020; Deolindo et al. 2020; Duda et al. 2024). In this study, theta therefore provided a clearer distinction between meditation and eyes‐closed rest than global alpha amplitude.

The alpha–SCL association nevertheless suggests that alpha activity remained related to sympathetic arousal during meditation. Comparable associations were not observed for delta, theta, beta, or gamma amplitude, arguing against a simple broadband relationship between EEG amplitude and sympathetic arousal. The positive association also differs from resting‐state findings in which greater global alpha activity has generally accompanied lower SCL (Barry et al. 2005, 2020). The relationship between alpha activity and sympathetic arousal may therefore be context dependent. During active breath‐focused meditation, greater alpha activity may coexist with higher sympathetic arousal rather than reflecting the lower‐arousal pattern typically observed during rest. This interpretation is consistent with previous work showing that alpha activity and sympathetic arousal can vary relatively independently during meditation (Duda et al. 2024, 2026; Duda, Clarke, and Barry 2025; Duda, Clarke, Barry, and De Blasio 2025). Rout et al. (2024) similarly found lower alpha activity and higher SCL during mind‐wandering than breath focus, without a significant association between their changes, suggesting that the two measures do not necessarily change in parallel across attentional states. Taken together, these findings suggest that alpha activity and sympathetic arousal are related in a context‐dependent manner during meditation, rather than following a fixed relationship across meditative and resting states.

The present findings show that physiological activity can change over relatively short periods within a single meditation session. Delta amplitude increased progressively across the meditation period, while SCL decreased initially before increasing later. Recent work has similarly identified time‐dependent changes in EEG activity over the course of meditation (Saketh et al. 2026), while experience‐sampling studies show that periods of breath focus and mind‐wandering are associated with different EEG profiles (Rodriguez‐Larios et al. 2021). Together, these findings highlight the potential loss of information when a meditation period is averaged into a single physiological estimate and support examining meditation as a temporally dynamic rather than uniform state.

The progressive increase in delta is more difficult to interpret. Delta activity has been associated with homeostatic and motivational processes during wakefulness (Knyazev 2012), and increases during some forms of meditation have previously been reported (Cahn et al. 2010; Dunn et al. 1999). It is therefore possible that the present increase reflects progressive settling into the practice or a greater inward focus of attention. However, declining vigilance provides an equally plausible explanation. Delta activity also increases with fatigue, sleepiness, and reduced wakefulness (Fujiwara et al. 2022; Knyazev 2012), and maintaining wakefulness is itself an important component of meditation (Britton et al. 2014). Because eyes‐closed rest always preceded meditation, meditation‐related change cannot be fully separated from elapsed time or declining vigilance. The SCL trajectory does not resolve this ambiguity, as sympathetic activity decreased initially but subsequently increased rather than showing a continuous decline. Appropriate comparison conditions are critical when attempting to isolate meditation‐specific effects from nonspecific effects of time and task context (Davidson and Kaszniak 2015; Van Dam et al. 2018), and suitable control conditions remain a recognized challenge in neurophysiological meditation research (Barrows et al. 2024). A duration‐matched eyes‐closed non‐meditation condition, ideally combined with independent measures of vigilance, would provide a stronger basis for interpreting these temporal changes.

The sex‐related findings were more limited. Males showed a larger theta increase from rest to meditation, while beta and gamma amplitudes were greater in females across states and beta also showed different temporal patterns in females and males during meditation. As the beta and gamma differences were present across conditions, they appear more consistent with general sex‐related variation in EEG amplitude than with meditation‐specific effects, in line with established sex differences in resting EEG (Cave and Barry 2021). Although beta activity has been associated with attentional engagement and alertness (Gola et al. 2013; Kamiński et al. 2012), this temporal sex difference was exploratory and occurred without an overall meditation‐related change in beta; its meaning therefore remains uncertain. The absence of a meditation‐related gamma effect should also be considered in light of differences in practitioner experience and the gamma frequency ranges examined across studies; for example, Braboszcz et al. (2017) reported elevated higher‐frequency gamma activity in experienced meditators relative to controls, whereas the present study examined 30.5–45.0 Hz activity in participants with limited meditation experience. The theta finding differed from the beta and gamma effects in reflecting a larger rest‐to‐meditation increase in males. However, the present sample had limited sensitivity for sex‐related interactions and this exploratory finding requires replication. The estimated alpha–SCL association was more positive in females than males, but the interaction was not significant, providing no reliable evidence of a sex difference. This exploratory pattern is of interest in light of evidence that women may derive greater psychological benefit from mindfulness training, with de Vibe et al. (2013) reporting improvements in mental distress, subjective well‐being, and study stress in women but not men. Whether these behavioral differences relate to the alpha–SCL pattern observed here remains unknown. Menstrual‐cycle phase, hormonal status, and contraceptive use were also not assessed, despite evidence that these factors can influence resting alpha activity and frequency (Bazanova et al. 2014, 2017; Brötzner et al. 2014), and may have contributed to variability within the female sample.

Several broader limitations should also be considered. Subjective measures of state mindfulness, mind‐wandering, sleepiness, attentional engagement, and perceived meditation depth were not obtained, limiting the extent to which the observed physiological changes can be linked to participants' moment‐to‐moment experience. First‐person experience is particularly relevant when interpreting meditation‐related physiology (Davidson and Kaszniak 2015), and studies incorporating experience sampling have demonstrated physiologically distinct periods of breath focus and mind‐wandering during meditation (Rodriguez‐Larios et al. 2021; Rout et al. 2024). More detailed assessment of participants' subjective experience has therefore been recommended to better characterize variation in meditative states (Barrows et al. 2024).

Respiration was also not measured. This is particularly relevant for breath‐focused meditation because breathing can influence both neural oscillatory activity and sympathetic arousal. Deliberate paced breathing has been shown to produce EEG and SCL responses that differ from mindfulness meditation despite both involving attention to breathing (Duda, Clarke, and Barry 2025), while intentional slow breathing during mindfulness meditation produces different neural and SCL responses from meditation involving spontaneous breathing (Duda et al. 2026). Without concurrent respiratory measurement, spontaneous changes in breathing cannot be separated from attentional or meditation‐related contributions to the present physiological effects. Recording respiration alongside EEG and SCL would therefore provide a clearer account of the processes underlying both neural and autonomic changes during breath‐focused meditation.

The brief meditation period and the limited meditation experience of the sample also constrain generalizability. Meditation experience has been associated with differences in both subjective experience and EEG activity during breath‐focused meditation (Rodriguez‐Larios et al. 2021), while broader reviews indicate that neurophysiological findings vary with practitioner expertise and meditation practice (Barrows et al. 2024; Lomas et al. 2015). Moreover, the temporal effects observed here and in other within‐session studies (Saketh et al. 2026) suggest that physiological characteristics measured during a brief period of practice should not necessarily be assumed to generalize to longer meditation sessions. Future work across different levels of experience and longer periods of practice would help determine which features are specific to early novice meditation and which generalize more broadly. The examination of multiple frequency bands, temporal patterns, and sex‐related effects also increases the possibility of chance findings among the exploratory analyses, which should therefore be considered preliminary until replicated in larger samples.

In conclusion, brief breath‐focused mindfulness meditation was characterized by increased theta activity without a marked overall change in SCL, together with a positive association between alpha activity and sympathetic arousal during meditation. Together, these findings are broadly consistent with relaxed alertness, suggesting a dynamic state in which attentional engagement is maintained without a sustained shift in sympathetic arousal. Exploratory findings, including the progressive increase in delta activity and sex‐related patterns, warrant further investigation using designs that can more clearly distinguish meditation‐specific effects from changes associated with time, vigilance, respiration, and individual differences.

## Author Contributions

**Alexander T. Duda:** conceptualization, methodology, software, data curation, investigation, validation, formal analysis, visualization, project administration, writing – original draft, writing – review and editing. **Adam R. Clarke:** conceptualization, methodology, formal analysis, supervision, resources, writing – review and editing. **Robert J. Barry:** conceptualization, methodology, formal analysis, supervision, writing – review and editing.

## Funding

The authors have nothing to report.

## Ethics Statement

This study was approved by the joint University of Wollongong/Illawarra Shoalhaven Local Health District Human Research Ethics Committee, protocol number 2020/363. All procedures were performed in accordance with relevant institutional guidelines and applicable ethical standards.

## Consent

Written informed consent was obtained from all participants prior to participation, and participant confidentiality was maintained.

## Conflicts of Interest

The authors declare no conflicts of interest.

## Data Availability

The datasets generated and/or analyzed during the current study can be obtained from the corresponding author upon reasonable request.
